# Non-contact injury incidence in professional women’s football depends on the starting status of the player

**DOI:** 10.5114/biolsport.2025.145916

**Published:** 2025-01-20

**Authors:** Victor Moreno-Perez, Berta Carles Bové, Javier Courel-Ibáñez, Juan Del Coso, Marco Beato, Eva Ferrer Vidal-Barraquer, Gil Rodas Font

**Affiliations:** 1 Sports Research Center, Miguel Hernandez University of Elche, Alicante, Spain; 2 Center for Translational Research in Physiotherapy. Department of Pathology and Surgery. Miguel Hernandez University of Elche, San Joan, Spain; 3 Sport Performance Department, FC Barcelona Sports, Barcelona, Spain; 4 Department of Physical Education and Sports, Faculty of Sport Sciences, Granada, Spain; 5 Sport Sciences Research Centre, Rey Juan Carlos University, Fuenlabrada, Spain; 6 School of Allied Health Sciences, University of Suffolk, Ipswich, United Kingdom; 7 Medical Department of Futbol Club Barcelona, FIFA Center of Excellence, and Barça Innovation Hub Barcelona, Spain

**Keywords:** Load monitoring, Performance, Tracking, Soccer, Team sports, Elite athlete

## Abstract

This study aimed to determine differences in the incidence of non-contact injury in professional women football players with different starting statuses. Data from 37 women (age: 28.5 ± 3.9 years; body mass: 62.3 ± 5.2 kg; height: 169.8 ± 4.7 cm) from a professional football team (Professional Women’s Football League from Spain) were prospectively collected during two consecutive seasons. Players were classified according to their match starting status as starters (players with > 70% of matches in the starting lineup; n = 20) and non-starters (n = 17). External load parameters were collected using GPS in all training sessions and matches. Non-contact injuries were diagnosed, classified, and recorded by the medical staff following the IOC consensus. Statistics included comparisons of starters vs. non-starters and linear regression and diagnostic analyses of injured vs. non-injured players. Non-starters accumulated less match load over the season but had two-fold non-contact injury incidence and three-fold muscular injury incidence during matches than starters, despite being exposed to a similar training load. The larger the number of matches played as a starter, the fewer injuries (non-contact: R^2^ = 0.27, p = 0.01; muscular: R^2^ = 0.11, p = 0.04). Diagnostic analyses identified clinical thresholds for insufficient match and training loads during the season accounting for higher injury risk. Less than 5,237 decelerations and 25 matches as a starter per season during training was the best indicator to discriminate players with less likelihood of non-contact injury. Football players with less match exposure from a women’s professional squad may be more prone to injury due to under-exposure to the demands of the game.

## INTRODUCTION

The popularity of women’s football has spiked over the last decade almost tripling the number of players, with more than 13 million women playing organized football worldwide [1, 2]. In parallel, the training and competition demands of women’s football have increased with a pronounced rise in the number of official matches per season and higher running distances during official matches, especially at high intensity [3, 4]. The increased physicality of women’s football has contributed to the current popularity of the game worldwide but it has also impacted football players’ health, particularly regarding an increased risk of injury [5]. Still, scientific evidence on women’s football remains sparse, counting for only up to 15% of the total number of football studies published in scientific journals [6]. A better understanding of specific match and training physical demands of women’s football over the season is needed to cope with some of the causes behind the progressive risk of injury in women’s football [7]. This knowledge would be key to adequately tailoring training to the competitive profile to maximize performance and minimize injuries [8]. Optimal training plans based on match demands would consequently increase the efficiency and effectiveness of football players during matches [9].

Much attention has been given to match physical demands in women’s football in the latest years [10–15]. Overall, previous research reported that outfield professional women football players typically cover distances ranging between ~8,200– ~11,000 m during official matches [11, 13, 16] of which ~300 m are above sprinting threshold [17]. Overall, women professional football players usually perform around 125 high-intensity actions per match [18]. Importantly, the referred external load values during official matches represent the most demanding session of the week [13], accounting for 12.13 ± 2.40 m · min^−1^ (~44.9% to 72.3%) of relative total distance covered and 1108 ± 294 m (~55.4% to 84.6%) of sprints during the week [13]. Accordingly, coaches should account for players’ match-time exposure and match load parameters to modulate the weekly loading patterns among all the players of the squad, particularly to compensate the loading differences between starters and non-starters [19–21]. The studies in women football players reveal that that players with higher involvement in the starting lineup over the season (i.e., starters) usually perform more time at high-intensity zones than non-starters [19, 20, 22]. In male football players, non-starters have lower match-time exposure over the season which implies impaired players’ physical fitness and performance [21, 23–25] and increased risk of muscle injury [26, 27]. Although professional football squads use personalized training strategies to compensate for the lower external load of non-starters [28], evidence in male football advocates for a diminished performance and increased risk of injury in non-starters as result of insufficient match loading. However, the performance and injury consequences of the under-exposure to the demands of the game non-starters in women’s football unknow.

Despite the increasing interest in women’s football, information on how to better manage match and training load remains little explored and requires clarification. Therefore, to determine differences in the incidence of non-contact injury in professional women football players with different starting status.

## MATERIALS AND METHODS

### Study design

This is a prospective cohort study examining match-time exposure, injury incidence, and match and training load of elite women football players competing in *Liga F* (Professional Women’s Football League from Spain) depending on their match starting status over the season. Overall, football players were classified according to their main starting status over the season as starters (players with > 70% of matches in the starting lineup). External load parameters were collected using GPS in all training sessions and matches over the season. This investigation was reviewed and approved by an institutional Ethics Review Committee.

### Participants

A total of 40 professional women football players (age: 28.5±3.9 years; body mass: 62.3 ± 5.2 kg; height: 169.8 ± 4.7 cm) from a squad of the Professional Women’s Football League from Spain (*Liga F*) were recruited and prospectively followed during two seasons (2019–2020 and 2020–2021). Match exposure comprised top football national and international competitions (Regular Season, Champions League, Copa de la Reina, and Super Cup). Inclusion criteria were: a) football players ≥ 18 years old; b) regular exercise training of > 1 h per day, > 5 days of training per week for the prior six months; c) training and competition with the first team of the club; d) participating in more than 10 matches during the season. Exclusion criteria were: a) players with a history of orthopaedic problems within the previous three months to the onset of the investigation; b) transfer to another club during the season. All players were fully informed about the study procedures and signed a written informed consent. Data were anonymized with an alphanumeric code before analysis.

### Injury data collection

Non-contact injuries were diagnosed, classified and recorded by the medical staff of the football team using the consensus statement for injury recording of the International Olympic Committee [30]. The medical staff for the team was previously instructed on how to report the information for each injury using a standardised form and specific definitions for the classification depending on the type of injury, body location and severity. Recorded injuries (region, body area, type of injury, tissue, pathology type, mode of onset [sudden and repetitive/gradual onset injuries]) were weekly collated via an electronic database. Specifically, the type of injury was reported after different medical confirmatory examinations (e.g., physical examination, X-ray, ultrasound, magnetic resonance imaging, etc). The mechanism of onset was also reported between contact and non-contact injuries was also collected and contact injuries were discarded as they are potentially unaffected by the different match exposure of the players. Based on the IOC statement, injuries were categorized about the events where they occurred as training and competition injuries. The days of absence from training and competition were recorded as a measure of injury severity [31]. Injury prevalence was calculated as the number of players who were diagnosed with a non-contact injury during the season. Injury incidence was individually calculated by dividing the number of injuries suffered by a player during the season by the player’s match and/or training exposure (injuries/1000 h).

### Match and training load

All players were monitored in all training and competition (matches) sessions using the same GPS 10-Hz portable system (Wimu ProTM, RealTrack Systems, Spain) [32]. Players’ training and competitive loads were monitored, registered and controlled by the team’s physical trainer, under the supervision of the head coach. Warm-up and between-halves match intervals were excluded. To avoid inter-unit error, each player wore the same GPS device during the whole study period [33]. The device was positioned inside a vest and it remained between the participants’ shoulders without hindering the upper body movements. Data were processed using the SPROTM software (RealTrack Systems, Almeria, Spain) [32]. Variables considered for the analyses were total running distance (m), high-speed running > 24 km · h^−1^, number of accelerations > 3 m · s^−2^, and decelerations < 3 m · s^−2^, in line with previous studies [34].

### Statistical analysis

Descriptive analyses included means, medians, frequencies and interquartile ranges (IQR) for each variable analysis. For each match within the season, players were categorized as starters if presented in the starting lineup and played > 60 min as this represents a high involvement in 2/3 of the total duration of the match. Then, we split the sample into two groups (starters and non-starters) according to the median of matches played as starters. Players over the median were classified as starters. Student’s t-test was used to identify mean differences in match and training load and injury incidence between starters and non-starters. The significance level was set at p < 0.05. Effect size (ES) was estimated by Cohen’s *d*, interpreted as small (0.20), medium (0.50) and large (0.80). Linear regression models were fitted to determine the relationship between the number of matches as a starter (considered as a continuous raw variable, i.e., the number of starting matches along the season) and match load, training load and injury outcomes in separate models. Regression coefficients (B) and standard error of the mean (SEM) were calculated to identify the relationships between variables. Receiver operating characteristics (ROC) curves were used to compare the diagnostic performance of each match and training load to classify injured and non-injured players). The true positive rate (Sensitivity) was plotted against the false positive rate (100-Specificity) for different cut-off points so that each point represents a sensitivity/specificity pair corresponding to a particular decision threshold. The area under the ROC curve (AUC) was calculated as a measure of how well each parameter can distinguish between the two diagnostic groups (injured/non-injured). The Youden index (*J*) and associated criterion were calculated, being *J* = 1 a perfect discriminatory ability. AUC > 0.70 and *J* > 0.30 were considered as a minimum level of acceptance [35]. Statistical calculations were performed using a custom Microsoft Excel spreadsheet, the SPSS v.28 (IBM Corp., Armonk, NY) and MedCalc v.18.2.1 (MedCalc Software bvba, Ostend, Belgium).

## RESULTS

Data from 37 players meeting the inclusion criteria were obtained (18 from season 2019–2020 and 19 from season 2020–2021). Three players were excluded due to having a recent injury (n = 1) and accumulating less than 10 games in the season (n = 2). Twenty players were classified as starters and 17 as nonstarters. The sample comprised 89 matches, 380 training sessions and 58 non-contact injuries (of these, 33 were muscular injuries). Severe injuries occurred in 17 players (46% of the sample), moderate injuries in 10 players (27%), mid injuries in 3 players (8%) and minimal injuries in 7 players (19%).

Starters described different training loads, match loads and injury incidences compared to non-starters (Table 1). Starters accumulated higher match loads, having lower match injury incidence per 1000 h (match non-contact injuries: 0.63 vs. 1.22 *p* = 0.022; match muscular injuries: 0.29 vs. 0.73, *p* = 0.028). In turn, training loads were very similar between starters and non-starters (*p* from 0.090 to 0.74).

**TABLE 1:** Accumulated external load parameters during training and matches and injury incidence of professional football players with different starting statuses over the season.

	Starters	Nonstarters	*p*	*d*
**Players (n)**	20	17		

**Matches (n)**	37.2 (8.1)	32.5 (7.0)	0.076	0.60

**Presence in the lineup (%)**	83.9 (8.5)	53.1 (10.5)	< 0.001*	3.24

**Training load**				
Duration (min)	8255 (1603)	7788 (2161)	0.457	0.25
Distance (km)	570 (128)	555 (138)	0.741	0.11
Running > 24 km · h^−1^ (m)	1905 (890)	2063 (1030)	0.620	-0.17
Accelerations > 3 m · s^−2^ (n)	4451 (825)	3942 (947)	0.090	0.58
Decelerations > 3 m · s^−2^ (n)	4796 (984)	4782 (1109)	0.968	0.01

**Match Load**				
Duration (min)	2694 (626)	1677 (423)	< 0.001*	1.87
Distance (km)	299 (82)	184 (68)	< 0.001*	1.50
Run > 24 km · h^−1^ (m)	2224 (1168)	1470 (1233)	0.065	0.63
Accelerations > 3 m · s^−2^ (n)	1263 (390)	728 (330)	< 0.001*	1.47
Decelerations > 3 m · s^−2^ (n)	1766 (532)	1140 (526)	0.001*	1.18

**Non-contact injury**				
Non-contact injuries (n)	1.30 (1.08)	1.88 (0.92)	0.090	-0.57
Training injury incidence (n/1000 h)	0.18 (0.16)	0.27 (0.17)	0.102	-0.55
Match injury incidence (n/1000 h)	0.63 (0.76)	1.22 (0.73)	0.022*	-0.79
Total injury incidence (n/1000 h)	0.12 (0.11)	0.2 (0.13)	0.053	-0.66

**Muscular injury**				
Muscular injuries (n)	0.65 (0.74)	1.11 (1.05)	0.124	-0.52
Training muscle injury incidence (n/1000 h)	0.08 (0.11)	0.17 (0.18)	0.105	-0.55
Match muscle injury incidence (n/1000 h)	0.29 (0.38)	0.73 (0.75)	0.028*	-0.76
Total muscle injury incidence (n/1000 h)	0.06 (0.08)	0.13 (0.14)	0.078	-0.60

Data are means and standard deviation, M(SD).; *d*= Cohen’s d effect size.

* Significant differences between groups (*p* < 0.05)

**FIG. 1:**
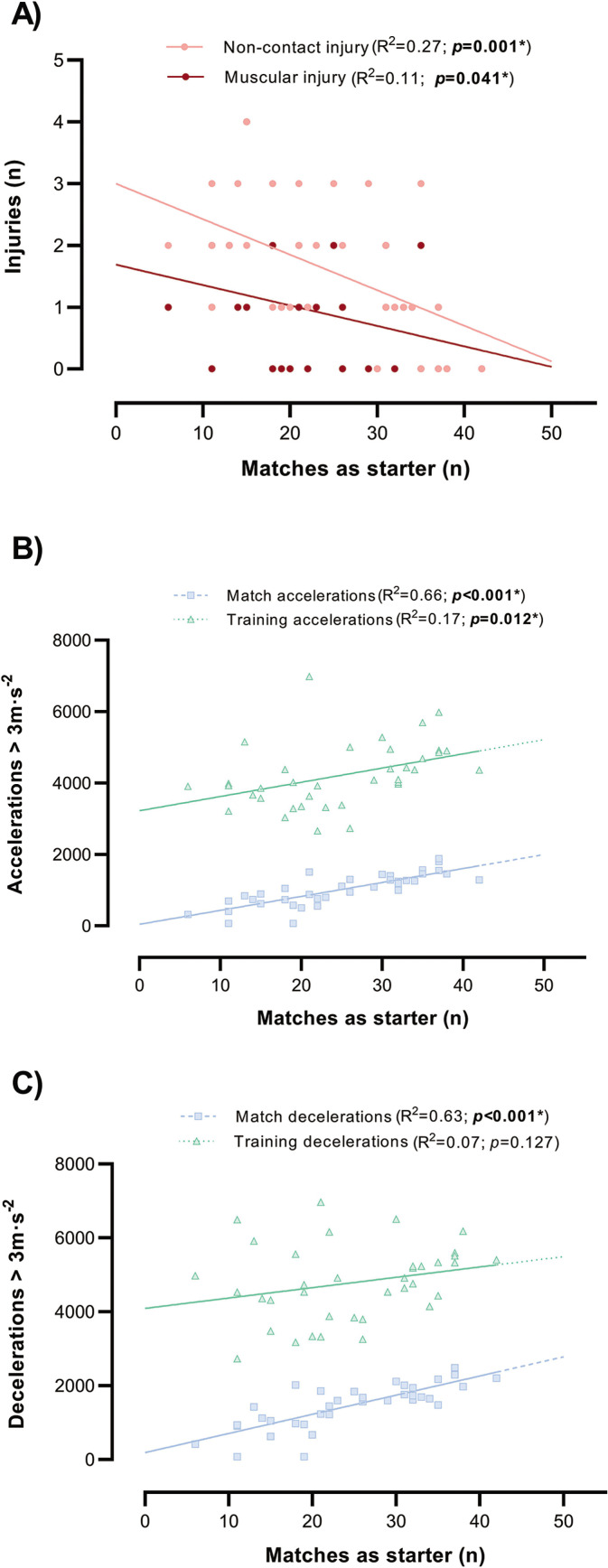
Linear relationships between seasonal matches as starter (X axis), number of non-contact and muscular injuries (Panel A), match and training accelerations (Panel B) and match and training decelerations (Panel C).

Regression analyses revealed a relationship between number of matches as starters over the season, the accumulated number of accelerations (either match and training) and the number of non-contact injuries (Figure 1). The larger the number of matches played as starter, the lower number of non-contact (R^2^ = 0.27, *p* = 0.001) and muscular injuries (R^2^ = 0.11, *p* = 0.041), the more training accelerations (R^2^ = 0.17, *p* = 0.012) and match accelerations (R^2^ = 0.66, *p* < 0.001) and decelerations (R^2^ = 0.63, *p* < 0.001).

**FIG. 2:**
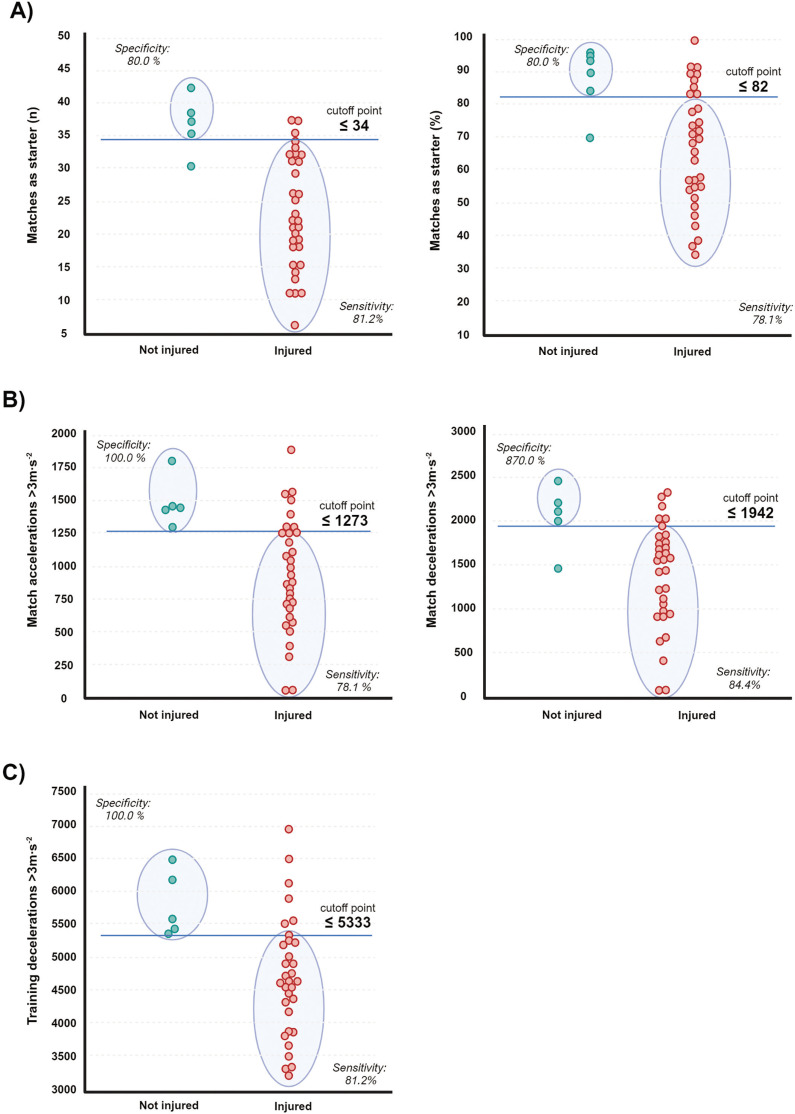
Aligned dot plot analyses and ROC derived threshold values to discriminate non-contact injuries by means of matches as starter (Panels A), training load (Panels B) and match load (Panels C).

**FIG. 3:**
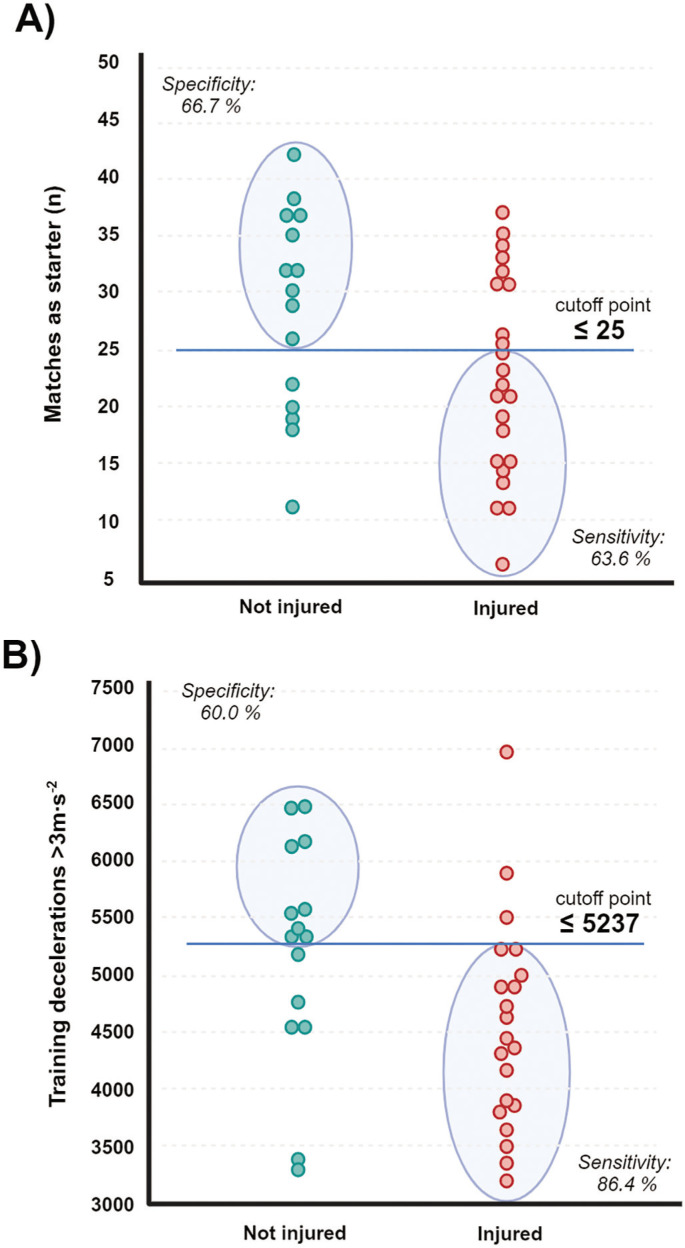
Aligned dot plot analyses and ROC derived threshold values to discriminate muscular injuries by means of matches as starter (Panel A) and training load (Panel B).

ROC analyses confirmed that seasonal matches as starter and training decelerations were significant indicators to discriminate between players with/without injury over the season. Thresholds for non-contact injuries were (Figure 2): Number of matches as starter ≤ 34 (AUC = 0.92, *p* < 0.001, J = 0.71), percentage of matches as starter ≤ 82 (AUC = 0.83, *p* < 0.001, J = 0.58), match acceleration ≤ 1273 (AUC = 0.78, *p* < 0.001, J = 0.78), match deceleration ≤ 1942 (AUC = 0.83, *p* < 0.001, J = 0.64), training acceleration ≤ 4096 (AUC = 0.88, *p* < 0.001, J = 0.66), and training deceleration ≤ 5333 (AUC = 0.88, *p* < 0.001, J = 0.82). Thresholds for muscular injuries were (Figure 3): Matches as starter ≤ 25 (AUC = 0.68, *p* = 0.042, J = 0.30) and training deceleration ≤ 5237 (AUC = 0.73, *p* = 0.010, J = 0.46).

## DISCUSSION

The present study aimed to determine differences in injury incidence in professional women football players with different match starting statuses exposure over the season. The data of this study showed that starters, as expected, accumulated higher match running distance, accelerations and decelerations over the season than nonstarters. However, players accumulated similar training loads regardless their starting status. This study is innovative in revealing for the first time that non-starters had a two-fold non-contact injury incidence and three-fold muscular injury incidence during matches than starters, despite being exposed to a similar training load (Table 1). The present results suggest that professional football players that participate in less than 70% of the matches of the season as a starter (~30±5 matches) are more prone to injury during matches. Although this study does not reveal the causes for this difference in the rate of injury between starters and nonstarters, players with less involvement in the starting lineup may likely be under-exposure to specific game demands, as previously reported for male football players [27]. Moreover, diagnostic analyses revealed that insufficient volume of accumulated training decelerations, in addition to the number of matches as starter, was the best discriminatory parameter to identify players at higher risk of non-contact injury and muscle-type injury.

Our findings seem to agree with earlier theories supporting the protective effect of players’ exposure to match play [27] or high-intensity running bouts [36] to help mitigate non-contact injury occurrences in elite men’s football. Data support the idea that engagement in actual game matches is the most effective way to prepare players for the physical challenges of match-play [19], and that consistent game participation throughout the competitive season is the most effective method for minimizing the chance of hamstring muscle injuries. Nonetheless, how to deal with non-starter players remains a challenge. The latest studies are seeking optimal workload programming during different turnarounds, suggesting that near-to-maximal sprinting speed may play a role in reducing non-contact injuries [36]. However, many questions remain unsolved and encourage future research to better understand how to deal with the problem of non-starters.

In line with our findings, previous research revealed that professional [19] and Under-23 [20] male starting football players and women collegiate starting players [37, 38] had higher match exposures [19, 20], covered longer running distance during the matches over the season, particularly at high-speed distance [19, 37, 38] and performed more accelerations and decelerations [20] compared to non-starters. Our results provide new information on training load and injury incidence depending on the starting status of the player. Interestingly, both starters and non-starters were exposed to a similar training load. In turn, non-starters had 61% less match exposure time and covered 63% less running distance over the matches than starters. Collectively, these data suggest that the training sessions were not effective in compensating non-starters for their lower participation in the matches, which probably affected their higher injury rate during real competitive situations. These results reinforce the need to introduce specific strategies to ensure that the non-starters receive similar weekly external load exposure to starters to be ready to cope with the demands of the game. The current substitution rules, with the possibility of introducing up to five substitutions, have provided a better frame for coaches to manage players’ match load but it is still needed to obtain weekly accumulated training and match load data to fully understand the stress imposed on the player [39].

Concerning the training load, the linear regression analysis revealed that those players with a higher number of matches as starters over the season also completed a higher number of accelerations during the training sessions. These data are counterintuitive as one may expect that players with higher involvement in official matches during the competitive season would present lower physical load during training, as they are habitually enrolled in recovery training sessions after the matches. A possible explanation for this result may be associated with the fact that the match represents the most demanding session of the week [13]. Hence, players with higher match-exposure times as a result of their habitual presence in the starting lineup may be more physically trained than their counterparts as it is probable that training does not reproduce the demands of the game. Another explanation may be associated with the different performance levels of the players, making coaches select those players who show better performance during training sessions more habitually to be part of the starting lineup. Independently of the reason, the current data unequivocally reveal that the physical load of non-starters during training does not compensate for their lower match exposure. Being under-loaded in official games, with no compensatory actions during training sessions, could be a mediator for higher injury rates in elite women football players.

Several external load thresholds were identified as hazardous to increase the risk of non-contact injury such as seasonal accumulated match acceleration ≤ 1273 and deceleration ≤ 1942, training accelerations ≤ 4096 and decelerations ≤ 5237, and the participation in less than 30 ± 5 (> 82%) matches as starter during the season. Most of the high-intensity accelerations and decelerations are the result of changes of directions and stoppages performed during the match [40], and are unlikely to be replicated during training settings. Accelerations and decelerations impose a high metabolic cost and muscle load demands [41, 42] due to their important eccentric muscular requirement [43], causing extensive damage to soft tissue structures [44, 45], decreased performance [46] and increased injury risks in football [47]. Paradoxically, while the accumulation of these high-intensity actions during a short period of a match may lead to a higher risk of non-contact injury, particularly muscle type and the ones located in the thighs [48, 49], our results suggest that being exposed to these actions over the season may produce a protective role against injury. Collectively, surpassing a certain (specific) load threshold of accelerations and decelerations during the season likely induced enhancements in fitness and tolerance to physical stress with a protective effect against non-contact injury. As a practical application, it seems essential that non-starters undergo acceleration and deceleration training tasks to compensate for the low match exposure, particularly by including training exercises that imply changes of directions and stoppages replicating those performed during the matches. The recording of the accumulated number of accelerations and decelerations over the season seems to be a key factor in identifying players at higher risk of injury.

## Limitations and future directions section

This study possesses several strengths associated with its novelty and the prospective nature of the analysis, the high-performance status of the sample and the standardized protocols employed to quantify match and training loads and record injuries. However, the current study is not exempt from research limitations; first, only one team was analysed in this study during the 2019–2020 and 2020–2021 seasons, therefore, the authors do not know whether the COVID period could have influenced the results. This team won the national championship in these two seasons and the UEFA Women’s Champions League in the 2020–2021 seasons. So, it is an elite team with a unique performance nature and it is possible that the results of this investigation, particularly the ones related to a threshold to discriminate between players with higher risk of injury do not apply to other teams, especially for those with lower competitive levels. A second limitation is related to the lack of quantification of the internal burden of load, such as mental fatigue, stress and perceptions of physical strain, which could offer additional information to football players [50]. This was due to the ecological approach used in this study that conformed to the teams’ strategies and the use of GPS to increase the robustness of the data-collection process [51]. Of note, players’ training intensity was adjusted based on the GPS records and the coach report, to offer compensatory work to avoid not-starter players having insufficient practice exposure. However, in the absence of training workload data, we cannot provide insights on how non-starters compensatory training may account for injury risk during the competition. Lastly, no information regarding coaches’ training strategies (i.e., post-game load compensation), dietary intake (e.g., stimulants that could have influenced their performance) or time of the day (i.e., how circadian rhythms affect the speed of conduction or muscular contractility) was recorded, which could have assisted in a better results interpretation, therefore future research could consider the monitoring of these variables.

## CONCLUSIONS

In summary, elite women football players with lower under-exposure to matches as a result of a lower involvement in the starting lineup over the season (i.e., nonstarters) had a higher incidence of non-contact injury than their counterparts with higher match involvement in the starting lineup (i.e., starters). The higher injury incidence in non-starters was probably associated with their lower accumulated match running distance and number of decelerations over the season which was not satisfactorily compensated during training. The accumulated number of training decelerations during the season stood as the best indicator to identify players with a higher likelihood of non-contact injury. These outcomes suggest that women football players with less involvement in the starting lineup may be more prone to injury due to under-exposure to the demands of the game. Being under-loaded in official games, with no compensatory actions during training sessions, could be a mediator for higher injury rates in elite women football players, as already reported in males. The match-time exposure accounts for non-contact injury incidence in women’s football. Greater match-time exposure reduces the injury incidence, which suggests that non-starter players receive insufficient load and require particular post-game training. A threshold for several matches as starter (less than 25–35) and training decelerations (less than 5300) accounted for greater muscular and non-muscular injury in both starters and non-starter players. The accumulated matches as starter seems a potential and practical indicator of injury risk. Therefore, underloaded players in terms of match exposure might need to be subjected to individual training strategies to minimize their potential risk of muscle injury.
